# A Collaborative Governance Strategy for Power Quality in AC/DC Distribution Networks Considering the Coupling Characteristics of Both Sides

**DOI:** 10.3390/s23187868

**Published:** 2023-09-13

**Authors:** Jiang Wang, Liang Qin, Yang Xiang, Penghui Ren, Xu Tang, Jiangjun Ruan, Kaipei Liu

**Affiliations:** 1 Hubei Key Laboratory of Power Equipment & System Security for Integrated Energy, Wuhan 430072, China; wangjangi@whu.edu.cn (J.W.); yangxiang@whu.edu.cn (Y.X.); w.phren@whu.edu.cn (P.R.); tangxu@whu.edu.cn (X.T.); ruan308@126.com (J.R.); kpliu@whu.edu.cn (K.L.); 2 School of Electrical Engineering and Automation, Wuhan University, Wuhan 430072, China

**Keywords:** AC and DC distribution networks, power quality, coupling mechanism, regional division, collaborative governance

## Abstract

The interactions between power quality in the AC-DC distribution network segments contribute to the distributed propagation of power quality anomalies throughout the entire network. Focusing on the photovoltaic multifunctional grid-connected inverter (PVMFGCI), this study deeply explores a collaborative governance strategy for optimizing regional power quality. Initially, the analysis dissects the DC ripple generation mechanism corresponding to harmonics and asymmetry in AC subnetwork voltages. Subsequently, a strategy is proposed for partitioning comprehensive control regions for AC-side power quality, taking into account photovoltaic governance resources based on insights from photovoltaic control realms and power quality classifications. Further, a collaborative allocation model for governance resources incorporating active optimization potentials of grid-connected converters is established based on the governance capabilities and residual capacities of PVMFGCI. Finally, the effectiveness of the proposed approach is validated through a MATLAB-based example analysis.

## 1. Introduction

With the increasing integration of distributed energy resources and demand-side inputs, the traditional unidirectional energy flow paradigm of distribution networks has evolved, giving rise to a dynamic landscape characterized by the coordinated orchestration of diverse energy streams [1]. As a result, the conventional structural framework of distribution networks is gradually transitioning towards the dynamic realm of AC and DC distribution networks [2]. Anchored in the concept of DC interconnection, the AC-DC distribution network assumes a pivotal role, serving not only as a conduit for the direct integration of wind and solar power to facilitate widespread utilization of distributed renewable energy, but also as an enabler of dynamic substation expansion and rapid fault remediation through the flexible medium of DC interconnection. This transformative shift represents an inevitable trajectory for the future development of distribution network infrastructure [3,4]. Within this evolving landscape, the inherent variability of photovoltaic active output, combined with the uncertainties inherent in single-phase grid-connected low-power distributed energy discharge, presents a propensity for power quality challenges. These challenges manifest in the form of AC-side harmonic injection, voltage oscillations, and tripartite imbalances, further escalating the stakes to encompass potential voltage excursions, system oscillations, and other safety concerns that have a tangible impact on the daily lives of the majority of users [5]. Moreover, the widespread proliferation of distributed power sources and power electronic devices amplifies the pervasiveness of power quality issues, embodying characteristics of high-penetration integration, decentralization, and comprehensive network assimilation. Given this complex scenario, a compelling mandate arises for comprehensive research aimed at the collaborative governance of decentralized, network-wide power quality issues within the realm of AC-DC distribution networks.

Presently, the complexity of power quality pollution distribution within AC and DC distribution networks is pronounced, and the consequential interactive effects accentuate the challenges posed to its governance. Notably, a spectrum of issues such as voltage harmonics, voltage deviations, and tripartite imbalances frequently plagues AC distribution networks. Conversely, the preeminent power quality concern in DC distribution networks revolves around the phenomenon of ripple, characterized by multifarious origins and far-reaching ramifications [6]. Within the milieu of AC-DC hybrid distribution networks, an intriguing interplay emerges where the transient power influx into the DC bus may carry inherent ripple components. This dynamic interjection can culminate in ripple occurrences within the DC bus voltage. In severe scenarios, this phenomenon reverberates to compromise not only power quality but also the overall dependability of the system’s power provision [7,8]. The literature [9] reveals the dominance of incorporating DC lines in regulating tie-line power oscillations compared to implementing AC lines alone. The literature in [10] advances a perspective from the vantage point of inverters, illuminating how the asymmetry inherent in AC loads can instigate voltage and current fluctuations within the DC capacitor, ultimately catalyzing ripple occurrences. Expanding this discourse, the literature in [11] encapsulates the primary triggers behind DC bus voltage ripple, attributing it to factors such as AC grid voltage asymmetry or grid voltage harmonics that give rise to imbalanced power components within the AC-DC interconnection power. Moreover, this intricate power quality interplay extends to encompass tripartite imbalances induced by power generation units or AC loads situated within the precincts of the DC distribution network. While the aforementioned power quality coupling mechanisms have been subject to meticulous analysis and derivation, endeavors encompassing the comprehensive control of power quality within distribution networks, anchored in the AC-DC coupling mechanism, remain relatively scarce.

For the power quality problems of AC and DC distribution networks, common solutions include installing active power filters (APF), static var generators (SVG), unified power quality regulators (UPQC), and other compensation devices. Among them, the voltage detection APF takes the node harmonic voltage as the control target, and calculates the APF compensation current according to the detection voltage and the set conductance value to suppress the voltage distortion of its access point [12,13]; SVG can realize dynamic compensation at the same time as reactive power and three-phase imbalance [14]; UPQC introduces intelligent algorithms based on football league algorithms into hybrid controllers to reduce voltage harmonics, improve power factor, and improve voltage imbalance [15,16,17] with better performance. At the same time, there have been many studies on the active optimization of power quality. The literature in [18,19] realized the use of inverter-type distributed generation (DG); the grid-connected nodes of the microgrid perform power quality control, integrate the grid-connected function and the APF function, and realize harmonic control and reactive power compensation while completing the DG active power grid-connected in the reference [20,21,22] for different situations. Application requirements introduce corresponding control strategies to improve power quality in terms of harmonic compensation, reactive power decentralized management, and load imbalance compensation, and at the same time realize grid connection of active power. The literature in [23] combines the voltage regulation capability of the photovoltaic power generation system with the electrical distance as the partition index, and proposes a double-layer voltage control strategy based on distribution network partitions. To sum up, in the existing treatment plan, adding compensation devices wastes photovoltaic treatment resources, and the active optimization of grid-connected inverters does not take into consideration its collaborative treatment in the whole grid power quality problem. Therefore, it is of great significance to study the active optimization and collaborative governance strategies of photovoltaic grid-connected inverters for the power quality of distribution networks.

According to the existing research, based on the typical grid structure of the AC-DC distribution network, this paper proposes a collaborative governance strategy for the power quality of the AC-DC distribution network based on the coupling characteristics of both sides. Its main potential has two points:

(i) Consider the photovoltaic grid-connected inverters in the distribution network to divide the power quality comprehensive management area of the AC and DC distribution network, which is conducive to the regionalization of power quality management.
(ii) Use the remaining capacity of the grid-connected inverter to manage the power quality of the whole network, and realize the reasonable and effective allocation of its governance resources so as to realize the global coordinated management of power quality.

## 2. Mechanism of DC Ripple Generation Based on AC-DC Coupling Characteristics

**Figure 1:**
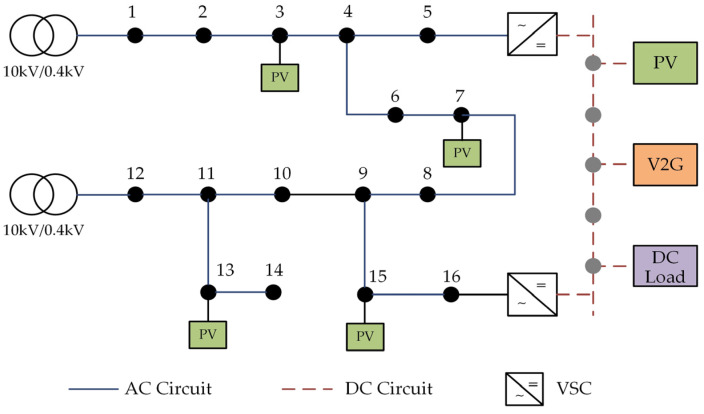
Topology of AC and DC distribution network.

Illustrated in Figure 1 is an exemplar of the typical AC-DC distribution network architecture. The comprehensive system is systematically categorized into discrete AC subnetworks, DC subnetworks, and converters. The AC subnetwork encompasses an array of AC loads and dispersed photovoltaic installations. In conjunction, the DC subnetwork and Voltage Source Converters (VSCs) collaboratively facilitate the dynamic integration of low-voltage AC and DC distribution networks. This adaptable interplay underscores the efficacy of accessing DC loads and engaging in photovoltaic energy storage in a manner that is both streamlined and efficient.

In the realm of the AC-DC distribution network, the presence of grid voltage harmonics or asymmetry on the AC side triggers a consequential interplay of unbalanced power components within the AC-DC interconnection power. This intricate interplay gives rise to the propagation of ripples onto the DC side, subsequently inducing fluctuations in voltage and current along the DC bus. The repercussions of these perturbations exert a pronounced impact on the overall power quality of the distribution network. Leveraging the coupling effect interdependence inherent to the power quality dynamics within the AC-DC distribution network, there emerges a compelling impetus to delve deeper into the mechanics underpinning the generation of ripples on the DC side. To address this imperative, the present paper undertakes a meticulous analysis of the mechanism, scrutinizing it through the lens of voltage harmonics and asymmetry embedded within the AC subnetwork [10].

### 2.1. Ripple Analysis Caused by Voltage Harmonics

In the presence of harmonic components within the voltage of the AC subnetwork, the voltage and current therein may be characterized as follows:

(1) $$\left\{\begin{array}{l} u_{a}=U_{m}\cos\left(\omega t-\varphi_{u}\right)+\sum_{k}U_{km}\cos\left(k\omega t+\theta_{ka}\right)\\ u_{b}=U_{m}\cos\left(\omega t-2\pi /3-\varphi_{u}\right)+\sum_{k}U_{km}\cos\left(k\omega t+\theta_{kb}\right)\\ u_{b}=U_{m}\cos\left(\omega t+2\pi /3-\varphi_{u}\right)+\sum_{k}U_{km}\cos\left(k\omega t+\theta_{kb}\right) \end{array}\right.$$

(2) $$\left\{\begin{array}{l} i_{a}=I_{m}\cos\left(\omega t-\varphi \right)\\ i_{b}=I_{m}\cos\left(\omega t-2\pi /3-\varphi \right)\\ i_{c}=I_{m}\cos\left(\omega t+2\pi /3-\varphi \right) \end{array}\right.$$

where *k* signifies the harmonic order, while $\theta_{ka}=0$, $\theta_{kb}=-2k\pi /3+\theta$, and $\theta_{kb}=-2k\pi /3+\theta$ denote the respective phases of distinct harmonic voltages.

Subsequently, the three-phase active power of the AC subnetwork can be deduced as follows:

(3) $$P_{AC}=P_{a}+P_{b}+P_{c}=P_{d}+\tilde{P}$$

where *P*_a_, *P*_b_, and *P*_c_ symbolize the three-phase powers of AC subnets A, B, and C, respectively. Meanwhile, Pd denotes the steady-state power quantity, and $\tilde{P}\;$ represents the power fluctuation engendered by harmonics.

The power within the *DC* subnet can be equationed as follows:

(4) $$P_{DC}=\frac{U_{dc}^{2}}{R_{dc}}+U_{dc}C_{dc}\frac{d{\tilde{u}}_{dc}}{dt}$$

where *R*_dc_ signifies the resistance on the *DC* side, *U*_dc_ represents the steady-state *DC* voltage, signifies the fluctuation quantity within the *DC* voltage, and *C*_dc_ denotes the capacitance value pertaining to the *DC* side.

The steady-state quantity and fluctuation quantity of power on both sides of the AC-DC distribution network are inherently equivalent. Thus, the ripples on the DC side can be calculated by utilizing the fluctuation quantity:

(5) $$\begin{array}{l} {\tilde{u}}_{dc}=\int \frac{\tilde{P}}{U_{dc}C_{dc}}dt\\ =\frac{1}{U_{dc}C_{dc}}\cdot \left[\int \sum_{k}U_{km}I_{km}(\sum_{h=0,\pm (k-1)}\cos((k-1)\omega t+\frac{2h\pi }{3}+\theta +\varphi ))dt+\int \sum_{k}U_{km}I_{km}(\sum_{h=0,\pm (k+1)}\cos((k+1)\omega t+\frac{2h\pi }{3}+\theta -\varphi ))dt\right] \end{array}$$

With reference to the preceding equation, it is discernible that the presence of a k-order harmonic in the AC subnetwork voltage gives rise to an (k±1)-order ripple within the DC subnetwork voltage.

### 2.2. Ripple Analysis Caused by Voltage Asymmetry

The imbalanced operation of the AC power distribution system engenders three-phase disparities in the grid voltage. Within the AC-DC distribution network, imbalanced loads on the AC side result in the generation of uneven components of voltage and current. This paper employs the symmetrical component method, wherein the composition of phase A current, comprising diverse sequence components, is expressed as follows:

(6) $$i_{a}=I_{m1}\cos(\omega t-\varphi_{1})+I_{m2}\cos(\omega t-\varphi_{2})+I_{m0}\cos(\omega t-\varphi_{0})$$

where *I*_m1_ and $\varphi_{1}$ denote the amplitude and phase, respectively, of the positive sequence component of the current. Similarly, *I*_m2_ and $\varphi_{2}$ represent the amplitude and phase of the negative sequence component of the current, while *I*_m0_ and $\varphi_{0}$ signify the amplitude and phase of the zero sequence component of the current, respectively.

(7) $$i_{dc}=\frac{1}{2}\left(S_{a}i_{a}+S_{b}i_{b}+S_{c}i_{c}\right)=\frac{3}{4}FI_{m1}\cos\left(\varphi_{1}\right)+\frac{3}{4}FI_{m2}\cos\left(2\omega t-\varphi_{2}\right)$$

Equation (7) reveals a direct correlation between the current and variables *F*, *I*_m1_, *I*_m2_, $\varphi_{1}$, and $\varphi_{2}$. Under circumstances of symmetrical three-phase load on the AC side, the absence of negative sequence component *I*_m2_ obviates any secondary ripple, maintaining a constant DC side current. Conversely, in cases of asymmetrical load, the DC side current encompasses not only the DC current but also twice the power frequency component. The introduction of a negative sequence component within the AC distribution network induces power oscillations, and it is noteworthy that the extent of load imbalance directly corresponds to the amplitude of the ripple on the DC side.

In summary, voltage harmonics and three-phase voltage imbalance within the AC subnetwork of the AC-DC distribution network instigate ripples within the DC bus, ultimately compromising the overall power quality and system reliability, particularly under critical conditions. Capitalizing on the DC ripple generation mechanism rooted in the AC-DC coupling characteristics, a collaborative governance strategy for the AC subnet emerges as a potent means to enhance active power stability, augment the quality of current entering the DC subnet, and effectively mitigate DC voltage ripple.

## 3. AC Side Power Quality Zoning Strategy Considering Distributed Photovoltaic Governance Resources

Photovoltaic grid-connected inverters exhibit the capacity to concurrently accomplish tasks such as grid-connected power generation, filtering, and reactive power compensation. Within the context of preserving active power generation, the surplus potential inherent in widely dispersed inverters can be effectively channeled towards power quality control. This entails a thoughtful incorporation of distributed photovoltaic management resources, leading to the establishment of distinct control zones. Such an approach fully capitalizes on the power quality management capabilities inherent in inverters within the distribution network.

### 3.1. Comprehensive Model for Voltage Sensitivity

Based on the presence or absence of photovoltaic grid connections at each node within the AC-DC distribution network, two distinct categories of grid nodes emerge: controllable source nodes and load nodes. Notably, controllable source nodes predominantly encompass the PV grid connection points. The prioritized segmentation of controllable source nodes results in each one forming an independent area. Subsequently, the control degree of controllable source nodes over all load nodes across the power grid is calculated, and load nodes exhibiting a substantial degree of control that surpasses the predetermined threshold are allocated to the respective controllable source area. This allocation method determines the control area for each photovoltaic unit, optimizing the influence of controllable source nodes on load nodes and ensuring effective management of photovoltaic units across the region. This study introduces a comprehensive voltage sensitivity model grounded in harmonic, reactive power, and three-phase imbalance sensitivities. Through this model, a partitioning strategy for photovoltaic control areas is devised which aptly considers the three primary power quality indicators.

#### 3.1.1. Harmonic Voltage Sensitivity

The harmonic voltage at each node in the distribution network is the aggregate of the harmonic voltage produced by individual harmonic sources at that node, alongside the harmonic compensation voltage generated by the photovoltaic grid-connected inverter. This relationship is expressed as follows:

(8) $$\left\{\begin{array}{l} U_{h}=U_{XIE.h}+U_{APF.h}\\ U_{APF.h}=Y^{-1}\cdot I_{APF.h} \end{array}\right.$$

where *U*_h_ represents the h-order harmonic voltage vector of the distribution network; *U*_XIE.h_ signifies the h-order harmonic voltage vector originated from the harmonic source; *U*_APF.h_ denotes the h-order harmonic compensation voltage vector produced by the photovoltaic grid-connected inverter; *Y*^−1^ represents the inverse matrix of the h-order harmonic admittance matrix of the distribution network; and *I*_APF.h_ indicates the h-order harmonic compensation current vector generated by the photovoltaic grid-connected inverter.

The harmonic control sensitivity calculation equation of the photovoltaic grid-connected inverter from node *j* to node *i* is obtained by determining and superimposing the corresponding weight factors based on the proportion of each harmonic voltage in the node voltage of the distribution network. The equation is expressed as follows:

(9) $$m_{ij}=\sum_{h=2}^{H}\alpha_{hi}\frac{\partial U_{hi}}{\partial I_{APF.hj}}$$

where *m*_ij_ represents the harmonic control sensitivity of the photovoltaic grid-connected inverter from node *j* to node *i*. The parameter $\alpha_{hi}$ signifies the weight associated with the h-order harmonic voltage in node *i*.

#### 3.1.2. Reactive Sensitivity

The reactive power sensitivity involves the partial derivatives of the fundamental voltage amplitude with respect to reactive power. These derivatives can be derived by utilizing the Newton–Raphson power flow calculation method in polar coordinates, as outlined in the reference. Suppose the system encompasses *n* nodes, comprising m PQ nodes, one balanced node, and *n* – m − 1 PV nodes. The power equation of the system can be represented in the form of polar coordinates.

(10) $$\left\{\begin{array}{c} \Delta P_{i}=P_{i}-V_{i}\sum_{j=1}^{n}V_{j}(G_{ij}\cos\delta_{ij}+B_{ij}\sin\delta_{ij})\\ \Delta Q_{i}=Q_{i}-V_{i}\sum_{j=1}^{n}V_{j}(G_{ij}\cos\delta_{ij}-B_{ij}\sin\delta_{ij}) \end{array}\right.$$

Through linearization, the corrected equation can be expressed as follows:

(11) $$\left[\begin{array}{c} \Delta P\\ \Delta Q \end{array}\right]=-\left[\begin{array}{cc} H_{\left(n-1\right)\times \left(n-1\right)} & N_{\left(n-1\right)\times m}\\ M_{m\times \left(n-1\right)} & L_{m\times m} \end{array}\right]\left[\begin{array}{c} \Delta \delta \\ \Delta V \end{array}\right]=-\left[\begin{array}{c} \Delta \delta \\ \Delta V \end{array}\right]$$

Furthermore, the sensitivity of fundamental voltage to reactive power can be determined as follows:

(12) $$S_{ij}=\frac{\partial V_{i}}{\partial Q_{j}}={\left(\frac{\partial Q_{j}}{\partial V_{i}}\right)}^{-1}$$

where *V*_i_ and *Q*_i_ are the fundamental voltage of node *I* and the reactive power of node *j*, respectively.

#### 3.1.3. Three-Phase Imbalance Sensitivity

Imbalanced loads within low-voltage distribution networks can lead to the generation of negative sequence currents, subsequently resulting in imbalanced three-phase voltages. The sensitivity analysis for three-phase imbalance initially involves evaluating the extent of imbalance in three-phase current or voltage. Subsequently, the sensitivity of the three-phase voltage imbalance to compensation current is computed.

(13) $$VUF_{i}=\frac{U_{2i}}{U_{1}}\times 100\%$$

(14) $$B_{ij}=\frac{VUF_{i}-VUF_{i}^{\prime }}{I_{APF.j}}$$

where *VUF*_i_ and $VUF_{i}^{\prime }$ represent the voltage imbalance degree of node *i* before compensation and after compensation, respectively.

To achieve a comprehensive approach towards addressing harmonic voltage, voltage deviation, and three-phase voltage imbalance, a method is devised based on harmonic sensitivity $E_{n_{h},ij}$, reactive power sensitivity *S_ij_*, and three-phase imbalance sensitivity *B*_ij_. The standardization of each index value is applied to mitigate the impact of varying magnitudes. The comprehensive voltage sensitivity index, denoted as *W*_ij_, is established through a weighting method. The expression for this index is presented below:

(15) $$x_{i}^{\prime }=\frac{x_{i}-\min\left(X\right)}{\max\left(X\right)-\min\left(X\right)}\left(i=1,2,\cdots ,q\right)$$

(16) $$W_{ij}=\tau_{e}\sqrt{\frac{1}{N}\sum_{n_{h}=2}^{H}E_{n_{h},ij}^{\prime 2}}+\tau_{s}S_{ij}^{\prime }+\tau_{b}B_{ij}^{\prime }$$

where $E_{n_{h},ij}^{\prime }$, $S_{ij}^{\prime }$, $B_{ij}^{\prime }$ represent the normalized calculation of the sensitivity index, respectively, $\tau_{e}$, $\tau_{s}$, $\tau_{b}$ represent the corresponding degree of importance, and the specific calculation is as follows:

(17) $$\tau_{e/s/b}=\frac{\sum_{i=1}^{n}\Delta D_{THi/VDi/TBi}}{\sum_{i=1}^{n}\left(\Delta D_{THi}+\Delta D_{VDi}+\Delta D_{TBi}\right)}$$

where *D*_THi_, *D*_VDi_, and *D*_TBi_ symbolize the total harmonic distortion rate of voltage at node *i*, the absolute value of voltage deviation, and the absolute value of three-phase voltage imbalance, respectively. The variable $\Delta D_{THi/VDi/TBi}$ is indicative of instances where the three indicators at node *i* surpass the predetermined limit prior to the distribution network governance intervention.

### 3.2. Division of Power Quality Zones in AC Subnetworks

The fundamental principle of regional power quality management lies in utilizing the correlation between nodes to delineate distinct regions, thereby facilitating comprehensive and decentralized control within intricate networks. The distribution network is segmented into multiple power quality control zones, distinguished by strong interconnectivity between nodes. Owing to the robust interdependence among nodes within each zone, a pivotal control node emerges naturally. By addressing power quality issues at this central node, the mitigation of analogous problems across other nodes within the same region is notably enhanced. This approach enables a more significant impact on regional governance with optimized resource utilization.

**Figure 2:**
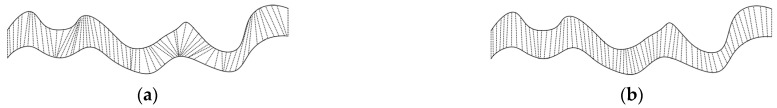
DTW and DDTW alignment. (**a**) DTW alignment. (**b**) DDTW alignment.

While Dynamic Time Warping (DTW) provides the capability to align sequences of unequal dimensions, akin to Edit Distance (ED), it does not distinctly depict the interplay between shape attributes and the directional evolution of the sequences. Additionally, it may exhibit a “sick bending” phenomenon characterized by substantial misalignments. This phenomenon pertains to the alignment of sequences with significant disparities. The Dynamic Derivative Time Warping (DDTW) algorithm is introduced to address these limitations and presents itself as a more apt choice for delineating power quality pollution zones compared to the DTW algorithm. The alignment diagrams for both methods are illustrated in Figure 2.

DDTW employs the directional change information inherent in the sequences for alignment matching, with the change trend being encapsulated by the first-order derivative. Consequently, within the DDTW matching algorithm, the comparison entails evaluating the squared difference of derivatives between each pair of points [24]. The procedural sequence is delineated as follows:

To preprocess the initial time series $X=\{x_{1},x_{2},\cdots ,x_{m}\}$ and $Y=\{y_{1},y_{2},\cdots ,y_{n}\}$, the differential technique is applied to transform their values, yielding $X^{\prime }$ and $Y^{\prime }$:

(18) $$X^{\prime }=\left\{\begin{array}{ll} {x^{\prime }}_{i}=\frac{(x_{i}-x_{i-1})+(x_{i+1}-x_{i-1})/2}{2} & ,\;2\leq i<m\\ {x^{\prime }}_{i}=x_{2}-x_{1} & ,\;i=1\\ {x^{\prime }}_{i}=x_{m}-x_{m-1} & ,\;i=m \end{array}\right.$$

(19) $$Y^{\prime }=\left\{\begin{array}{ll} {y^{\prime }}_{j}=\frac{(y_{j}-y_{j-1})+(y_{j+1}-y_{j-1})/2}{2} & ,\;2\leq j<n\\ {y^{\prime }}_{j}=y_{2}-y_{1} & ,\;j=1\\ {y^{\prime }}_{j}=y_{n}-y_{n-1} & ,\;j=n \end{array}\right.$$

Normalize the element values $X^{\prime }$ and $Y^{\prime }$ within sequences ${x^{\prime }}_{i}$ and ${y^{\prime }}_{j}$:

(20) $$\left\{\begin{array}{l} {x^{\prime }}_{i*}=\frac{{x^{\prime }}_{i}-\mu_{X^{\prime }}}{\sigma_{X^{\prime }}}\\ {y^{\prime }}_{j*}=\frac{{y^{\prime }}_{j}-\mu_{Y^{\prime }}}{\sigma_{Y^{\prime }}} \end{array}\right.$$

where $\mu_{X^{\prime }}$ and $\mu_{Y^{\prime }}$ are the mean values of $X^{\prime }$ and $Y^{\prime }$, respectively; $\sigma_{X^{\prime }}$ and $\sigma_{Y^{\prime }}$ are the variances of $X^{\prime }$ and $Y^{\prime }$, respectively.

After this preprocessing, the transformed sequences $X_{*}^{\prime }=\{{x^{\prime }}_{1*},{x^{\prime }}_{2*},\cdots ,{x^{\prime }}_{m*}\}$ and $Y_{*}^{\prime }=$ $\left\{{y^{\prime }}_{1*},{y^{\prime }}_{2*},\cdots ,{y^{\prime }}_{n*}\right\}$ are obtained, following which the DTW calculation is conducted to derive the DDTW distance.

The FCM (Fuzzy C-Means) algorithm incorporates the notion of membership, assessing the degree of affiliation of each sample to different categories through the membership matrix. The likelihood of a sample belonging to each category exists within the range of [0, 1]. Consequently, the FCM clustering algorithm represents a form of fuzzy soft clustering. The central concept revolves around iteratively refining the membership matrix and cluster centroids until the objective function achieves its minimum, ultimately yielding the definitive cluster centers.

Consider a sample dataset denoted as $X=\{x_{1},\cdots ,x_{j},\cdots ,x_{n}\}$, where *n* signifies the total count of sample data, and *j* represents the subscript associated with each sample datum.

The objective function of the FCM clustering algorithm is expressed as follows:

(21) $$\left\{\begin{array}{l} \min J(U,V)=\sum_{i=1}^{c}\sum_{j=1}^{n}\mu_{ij}^{m}d_{ij}\\ s.t.\mu_{ij}\in [0,1],\forall i,j;\sum_{i=1}^{c}\mu_{ij}=1,\forall j \end{array}\right.$$

where *c* represents the total number of cluster categories, *i* represents the subscript of the cluster category; $U=\{\mu_{ij}\}$ represents the c×n-order membership degree matrix, and $\mu_{ij}$ represents the membership degree of the *j*-th sample data belonging to the *i*-th class; $V=\{v_{i}\}$ represents the cluster center matrix of order c×d, and $v_{i}$ represents the cluster center of the i-th class; m∈[1,+∞) is the fuzzy index, *m* = 2; $d_{ij}=d_{\mathrm{DDTW}}(x_{j},v_{i})$ represents the DDTW distance from the sample $x_{j}$ to the cluster center $v_{i}$ of the *i*-th class.

The relationship linking the Euclidean distance between individual elements and the cluster center to the membership degree matrix is established through the utilization of Lagrange multiplication. This relationship, in turn, facilitates the derivation of an iterative link between the membership degree matrix and the cluster center. FCM, classified as an unsupervised algorithm, operates on sample data for which categorization is typically unknown. For effective application of the FCM algorithm, a specific cluster count c must be designated to segment the sample data set into c distinct categories. The foundational principle governing clustering algorithms underscores the goal of minimizing intra-class distances while concurrently maximizing inter-class distances. Within this principle, the number of clusters denoted by c holds significant influence. Thus, a function that employs the number of clusters c as an independent variable is essential to characterize clustering performance. This function is aptly termed the clustering validity function. This function serves to evaluate the efficacy of clustering by considering the ratio of inter-class distance to intra-class distance, quantified through the functional evaluation index *P* [25]. Notably, a higher value of *P* signifies a more favorable outcome in terms of clustering effectiveness, reflecting improved data separation and quality of categorization.

(22) $$P=\frac{\frac{1}{c}\sum_{i=1}^{c}\sum_{\begin{array}{l} k=1\\ k\neq i \end{array}}^{c}\frac{1}{4}\left(\max d_{\mathrm{FDDTW}}(v_{i},x_{j}^{\left(k\right)})+\min d_{\mathrm{FDDTW}}(v_{i},x_{j}^{\left(k\right)})\right)}{\sum_{i=1}^{c}\frac{1}{n}\sum_{j=1}^{n}d_{\mathrm{FDDTW}}(v_{i},x_{j}^{\left(i\right)})}$$

where $x_{j}^{\left(k\right)}$ signifies the *j*-th sample data that belong to the *k*-th class. The numerator in the expression represents the inter-class distance, while the denominator signifies the intra-class distance. Upon reaching its maximum value, the clustering validity index *P* aids in determining the optimal number of clusters, denoted as c, for the given sample data set.

Building upon the foundation laid out in Equations (16) and (17), the tripartite power quality indices encompassing harmonics, voltage deviation, and three-phase imbalance are meticulously weighted and amalgamated for every node situated within the expansive AC and DC distribution network. This intricate synthesis yields a unified power quality time series, which emerges as a potent tool for crafting an AC regional division strategy. This strategy, rooted in the fusion of power quality data, becomes the linchpin for orchestrating a comprehensive approach to managing power quality within subnetworks, ensuring their harmonious and effective operation.

### 3.3. Process of AC-Side Power Quality Zoning Strategy Considering Distributed Photovoltaic Governance Resources

**Figure 3:**
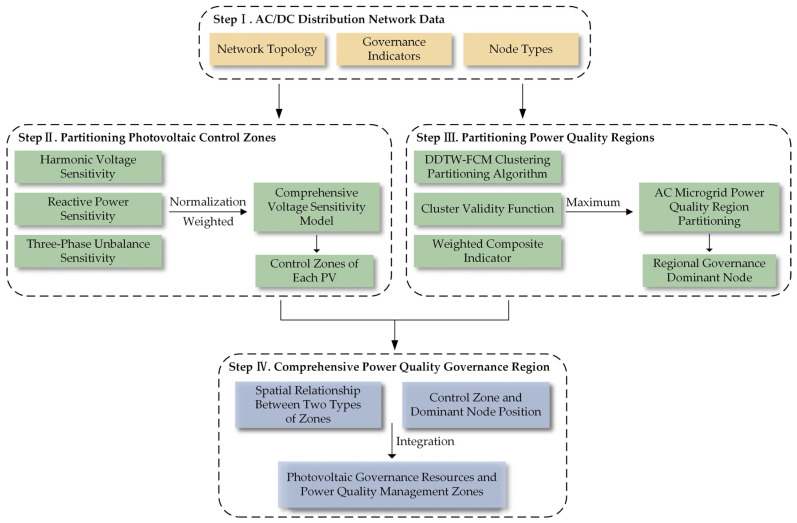
Process of Regional Division Strategy for Comprehensive Power Quality Management in AC and DC Distribution Networks.

This paper presents a comprehensive regional division strategy for managing power quality in AC and DC distribution networks. The process is illustrated in Figure 3, with detailed steps outlined as follows:

(i) A comprehensive voltage sensitivity model is equationed by integrating harmonic sensitivity, reactive power sensitivity, and three-phase imbalance sensitivity. This model takes into consideration the presence of distributed photovoltaic governance resources within the distribution network. It delineates photovoltaic control areas that effectively address the three primary power quality indicators.
(ii) The DDTW-FCM clustering and partitioning algorithm is harnessed to analyze time series data. This approach determines the optimal number of clusters through the maximization of the clustering validity function. Subsequently, it assigns nodes to respective classes, thereby facilitating the delineation of power quality areas. Each area’s central node, closest to the cluster center, is identified as the pilot node for regional governance.
(iii) The comprehensive management area of power quality is defined based on the spatial relationship between the two types of areas through the following methods: if the photovoltaic control area is entirely encompassed within the power quality area, the comprehensive management area is set to be the extent of the photovoltaic control area. In cases where the regions overlap, the approach involves amalgamating the PV control region with the corresponding power quality region of the predominant node it encompasses.

## 4. Optimal Allocation of Distributed Governance Resources Considering Active Photovoltaic Grid-Connected Converter Optimization

### 4.1. Functional Analysis of Power Quality Control in Photovoltaic Grid-Connected Inverters

**Figure 4:**
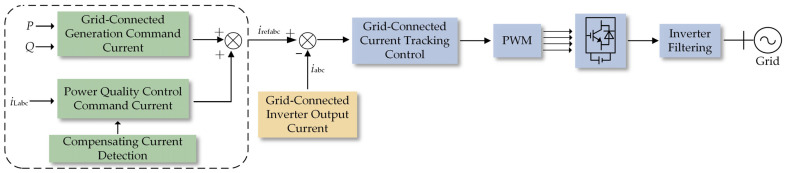
PVMFGCI system structure diagram.

In the low-voltage distribution network of my country, the prevalent power supply method often employs three-phase four-wire configurations [26,27]. The four-leg photovoltaic grid-connected inverter equipped with active filtering capabilities encompasses various technologies, such as grid-connected power generation, active filtering, Maximum Power Point Tracking (MPPT), command current calculation, and tracking control. The system’s structural diagram is illustrated in Figure 4. The primary circuit comprises components such as a photovoltaic array, a three-phase four-leg converter, filter inductors, parallel loads, and the three-phase four-wire power grid. The control loop integrates elements such as the signal detection unit, Maximum Power Point Tracking (MPPT) control unit, command current calculation unit, and the main control unit.

The control loop of the Photovoltaic Grid-Connected Inverter (PVMFGCI) continuously monitors the load current within the distribution network. It extracts harmonic, reactive power, and imbalanced current components, subsequently generating a reference value for compensation current. This compensation current reference is then integrated with the active current from photovoltaic power generation to establish the final grid-connected command current. This approach ensures cohesive regulation of power generation and active filtering functions during grid connection. The present study places emphasis on the synergistic utilization of photovoltaic grid connection and parallel-connected Active Power Filters (APF). This integrated approach is particularly effective in addressing power-related issues such as grid imbalances, all the while contributing active power to the grid. Due to the scope of this paper, detailed discussion on the Maximum Power Point Tracking (MPPT) technique is not provided.

The Photovoltaic Grid-Connected Inverter (PVMFGCI) has a total capacity denoted as *S*_N_. Among this capacity, a portion is allocated to DG grid connection, represented as *S*_DG_, leaving the remaining capacity designated as *S*_RE_.

(23) $$\left\{\begin{array}{l} S_{N}^{2}=S_{DG}^{2}+S_{RE}^{2}\\ S_{DG}=3UI_{1} \end{array}\right.$$

where *U* is the distribution network voltage, and *I*_1_ is the active current output by the inverter.

The interplay between the harmonic control and reactive power compensation capacity of the photovoltaic grid-connected inverter and its remaining capacity can be equationed as follows:

(24) $$\sqrt{S_{h}^{2}+S_{q}^{2}+S_{2}^{2}}\leq S_{RE}$$

where *S*_h_, *S*_q_, and *S*_2_ represent the capacities of the DG grid-connected inverter for harmonic control, reactive power compensation, and three-phase imbalance control, respectively.

### 4.2. Power Quality Optimization Strategy Utilizing Adjustable Capacity in Photovoltaic Grid-Connected Systems

Taking into account the geographical placement and capacity of distributed generation (DG) grid-connected inverters within the distribution network, a prudent allocation of residual inverter capacity can yield tailored governance outcomes, thereby differentially influencing power quality across distinct distribution network regions. Through the meticulous optimization of compensation strategies, the efficacy of the photovoltaic multifunctional grid inverter (PVMFGCI) in mitigating harmonics, reactive power, and three-phase imbalances can be rendered more cost-effective and logically coherent. This approach fosters an efficient distribution of governance resources, thereby achieving a heightened level of power quality enhancement across various sectors of the network, as elucidated in reference [28].

Building upon the allocated capacity of PVMFGCI, a mathematical framework is devised to determine the optimal compensation coefficients for harmonics, reactive power, and three-phase imbalances using its residual capacity. The subsequent equations serve to articulate these coefficients individually:

(25) $$\left\{\begin{array}{l} l_{h}=I_{h}/I_{1}\\ I_{h}=\sqrt{\sum_{n_{h}=2}^{H}I_{n_{h}}^{2}}\\ l_{h}=I_{q}/I_{1} \end{array}\right.$$

(26) $$\left\{\begin{array}{l} l_{h}=I_{h}/I_{1}\\ l_{q}=I_{q}/I_{1}\\ l_{0}=I_{0}/I_{1} \end{array}\right.$$

where *I*_1_, *I*_h_, *I*_q_, and *I*_0_ represent the fundamental current, harmonic current, reactive current, and three-phase imbalanced current on the grid side, respectively.

Suppose the coefficients for harmonic control, reactive power compensation, and three-phase imbalance control of the remaining capacity of PVMFGCI are represented as $\varepsilon_{h}$, $\varepsilon_{q}$ and $\varepsilon_{2}$, respectively. In this context, the power quality coefficient after compensation can be derived as follows:

(27) $$\left\{\begin{array}{l} l_{h}=\left(1-\varepsilon_{h}\right)l_{h0}\\ l_{q}=\left(1-\varepsilon_{q}\right)l_{q0}\\ l_{2}=\left(1-\varepsilon_{0}\right)l_{20} \end{array}\right.$$

where $l_{h0}$, $l_{q0}$, $l_{20}$ and $l_{h}$, $l_{q}$, $l_{2}$ are the harmonics, reactive power and three-phase imbalance coefficients before compensation and after compensation, respectively.

Developing a comprehensive evaluation model for power quality under various operating scenarios involves solving for PVMFGCI [29].

(28) $$\left\{\begin{array}{l} \min\left(l_{h}+l_{q}+l_{2}\right)\\ S_{out}=3U\sqrt{I_{p1}^{2}+I_{ph}^{2}+I_{pq}^{2}+I_{p2}^{2}}\\ 0\leq \varepsilon_{h},\varepsilon_{q},\varepsilon_{0}\leq 1 \end{array}\right.$$

where *I*_p1_, *I*_ph_, *I*_pq_, and *I*_p2_ are PVMFGCI output active current, harmonic wave control, reactive power compensation, and three-phase imbalance control current, respectively.

Considering the simplicity of the Lagrange multiplier method, this study employs this approach to solve the optimal coefficient problem related to the comprehensive power quality control of the remaining capacity of PVMFGCI [30]. Let *λ* represent the Lagrangian multiplier, and equation the Lagrangian function as follows:

(29) $$L_{G}=l_{h}+l_{q}+l_{2}+\lambda (I_{p1}^{2}+I_{ph}^{2}+I_{pq}^{2}+I_{p2}^{2}-\frac{S_{N}^{2}}{9U^{2}})$$

Compute using the Lagrange multiplier method in order to solve:

(30) $$\left\{\lambda =\frac{1}{2I_{1}}\sqrt{\frac{3}{\left(\frac{S_{N}^{2}}{9U^{2}}-I_{1}^{2}\right)}},\;\varepsilon_{h}=\frac{1}{l_{h0}I_{1}}\sqrt{\frac{\left(\frac{S_{N}^{2}}{9U^{2}}-I_{1}^{2}\right)}{3}},\;\varepsilon_{q}=\frac{1}{l_{q0}I_{1}}\sqrt{\frac{\left(\frac{S_{N}^{2}}{9U^{2}}-I_{1}^{2}\right)}{3}},\;\varepsilon_{2}=\frac{1}{l_{20}I_{1}}\sqrt{\frac{\left(\frac{S_{N}^{2}}{9U^{2}}-I_{1}^{2}\right)}{3}}\right\}$$

The comprehensive management strategy of power quality of PVMFGCI remaining capacity is as follows:

(i) A comprehensive voltage sensitivity model is equationed by integrating harmonic sensitivity, reactive power sensitivity, and three-phase imbalance sensitivity. This model takes into consideration the presence of distributed photovoltaic governance resources within the distribution network. It delineates photovoltaic control areas that effectively address the three primary power quality indicators.
(ii) If *S*_RE_ ≥ *Q*_N0_, and satisfies
  (31) $$3U\sqrt{I_{ph}^{2}+I_{pq}^{2}+I_{p2}^{2}}\leq S_{RE}$$

Subsequently, full compensation can be executed directly when the value of $\varepsilon_{h}=\varepsilon_{q}=\varepsilon_{0}=1$ is met. Conversely, if this criterion is not fulfilled, calculate the compensation coefficients using Equation (30) prior to implementing the compensation.

(iii) If *S*_RE_ ≤ *Q*_N0_, and satisfy, then no compensation will be invested.

## 5. Case Analysis

To validate the efficacy of the zoning management approach outlined in this study, we employ the AC/DC distribution network depicted in Figure 1 as a reference model. This network encompasses a 16-node 220 V AC subnet and a five-node ±375 V DC subnet, interconnected in the distribution system. The VSC (Voltage Source Converter) capacity is established at 200 kV·A, with photovoltaic (PV) integration accomplished through AC three-phase and DC connections. Notably, the analysis disregards the influence of harmonic absorption by system capacitors and assumes ideal transformers. The comprehensive management of power quality is exclusively undertaken using the PVMFGCI. At the grid-connected node, the PVMFGCI capacity is set at 18 kVA, and the solar cell array output is optimized for maximum power, attaining 15 kW. Despite allocating the full PVMFGCI capacity for grid connection and harmonic control, a certain surplus capacity remains untapped, which is neither utilized for active grid connection nor harmonic control. For calculation purposes, this available capacity is deemed to be 17 kVA in the ensuing example. Drawing from the typical daily active power output curve of photovoltaics, the designed harmonic control optimization strategy is substantiated through an illustrative case study.

Adhering to the harmonic standards applicable in my country, this section establishes a permissible upper threshold of 4% for the Total Harmonic Distortion Rate of harmonic voltage. The harmonic distribution among the system nodes, pertinent to the calculation example, is outlined in Table 1. Additionally, the harmonic current is taken into account at frequencies corresponding to the 5th, 7th, 11th, and 13th harmonics. Specifically, nodes 3, 7, 13, and 15 are associated with 20 kW imbalanced loads individually.

Utilizing the comprehensive voltage sensitivity model, it becomes possible to delineate photovoltaic control areas within the AC subnet, factoring in the three power quality indicators. These areas are established for each of the four photovoltaic sources, with their corresponding controllable source nodes and the nodes under their control outlined in Table 2.

As observed in the table, the four controllable source nodes align with distinct photovoltaic control areas. Given that power quality compensation takes the form of current, each control area predominantly encompasses nodes situated along the same branch. Under the function of photovoltaic power quality management, enhancements in power quality can be anticipated within the photovoltaic control area. Fundamentally, the compensation processes for harmonics, reactive power, and imbalance within the system all revolve around controlling the current waveform. The application of uniform compensation results in a system current that is both symmetrical and sinusoidal in nature.

**Figure 5:**
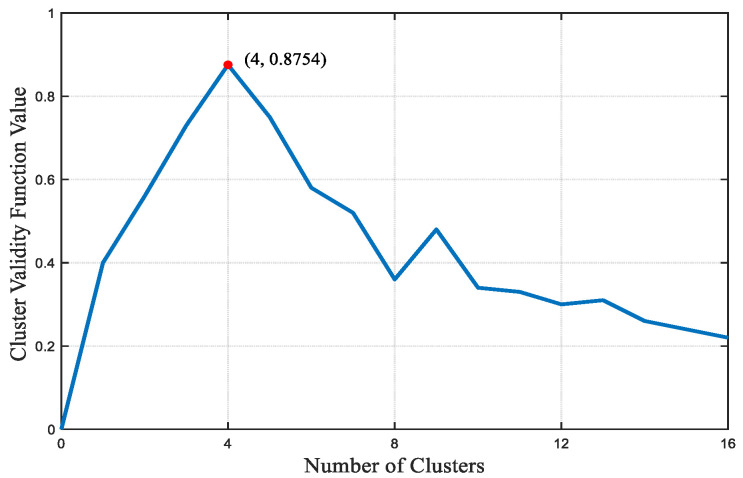
Clustering validity function value.

The power quality time series data from the 16 nodes undergo dimension reduction, followed by the application of the DDTW-FCM method for power quality partitioning. Within each partition, the node nearest to the cluster center is designated as the dominant node. The achieved clustering effectiveness is depicted in Figure 5, while the specific partition outcomes are presented in Table 3.

From the clustering validity function, it is evident that the optimal division of regions is achieved when the number of regions is four. At this point, the power quality coupling between regions is minimized, while the coupling within each region is maximized. Referring to the table, the dominant control nodes for regions I, II, IV, and V are identified as 3, 7, 15, and 11, respectively. Based on this regional division strategy for comprehensive power quality management in the AC and DC distribution network, a refined power quality division on the AC side, considering distributed photovoltaic management resources, can be further established.

**Figure 6:**
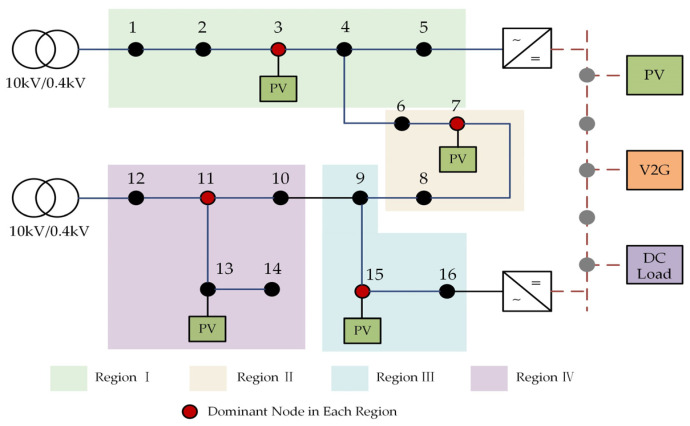
Regional Segmentation and Primary Nodal Points for Comprehensive Power Quality Management.

Drawing upon the spatial relationship between these two area types, the comprehensive power quality control areas are precisely delineated. Specifically, the AC subnetwork of the AC-DC distribution network is partitioned into four comprehensive power quality control areas, as illustrated in Figure 6. This approach aids in efficiently consolidating the governance resources of distributed photovoltaics, guaranteeing that photovoltaic control is harmonized to enhance power quality. Ultimately, it facilitates the optimal execution of comprehensive governance strategies.

Derived from the power quality optimization strategy involving the adaptable capacity of the photovoltaic grid-connected system, the optimal coefficients for compensating harmonics, reactive power, and three-phase imbalance are deduced. The resulting capacity allocation and compensation coefficients are illustrated in Table 4.

**Figure 7:**
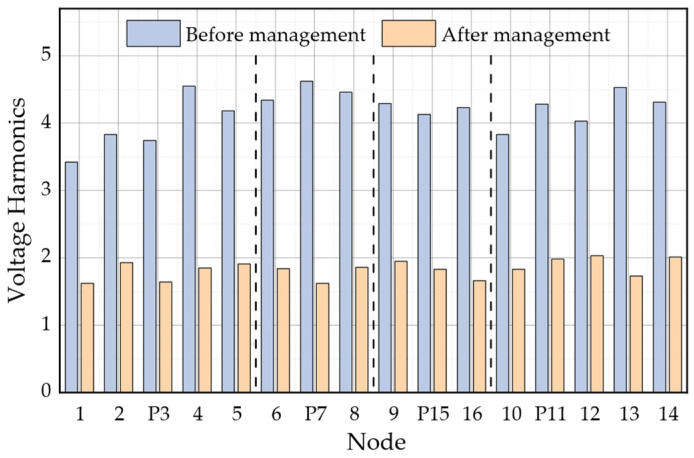
Harmonic Distortion Distribution Before and After Comprehensive Treatment in Different Areas.

**Figure 8:**
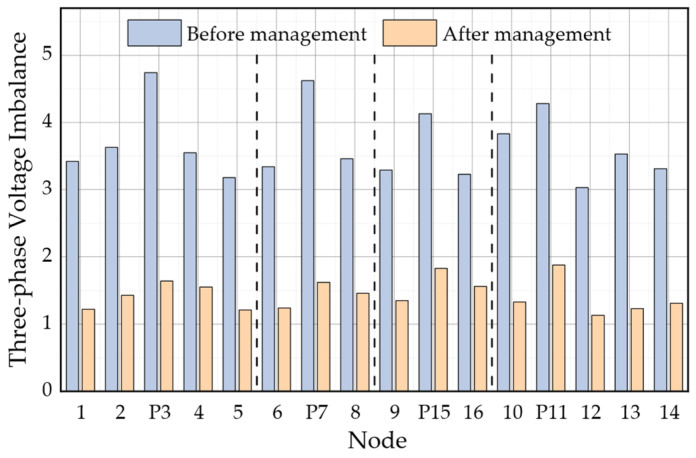
Three-Phase Imbalance Distribution Before and After Comprehensive Treatment in Different Areas.

As indicated in Table 4, the choice of 12:00 results in a maximum PV output power of 15 kW, and the computed power quality compensation capacity remains well within the confines of the remaining capacity. This enables a full and unabated active grid connection. The figures below, specifically Figure 7 and Figure 8, present a visual representation of the outcomes pertaining to harmonics and three-phase imbalance before and after the comprehensive power quality control for each node within the AC subnet of the AC and DC distribution network.

From the figure, it is evident that following the comprehensive treatment of the AC subnet, the harmonics at each node are consistently below 2%. Moreover, the degree of three-phase imbalance experiences a noticeable reduction, and the calculated ripple coefficient of the DC bus decreases from 1.75% to 0.5%. The implementation of power quality control measures within the AC subnet proves effective in diminishing the introduced ripple, enhancing the power quality of the DC subnet, and ensuring the stable operation of the DC subnet.

## 6. Conclusions

In order to improve the power quality of the AC-DC distribution network and give full play to the advantages of flexible and controllable output power of photovoltaic power generation, this paper proposes a collaborative governance strategy for the power quality of the AC-DC distribution network based on the coupling characteristics of both sides, and comprehensively considers the coupling characteristics of power quality, rationally allocates photovoltaic governance resources, and realizes the comprehensive optimization of power quality.

(i) In the problem of power quality on both sides of AC and DC, the DC ripple generation mechanism corresponding to the harmonics and asymmetry of the AC subnetwork voltage is derived and analyzed. With the help of the inverter’s fast response and high-precision adjustment capabilities, it can quickly identify and respond to current distortions and imbalances and achieve effective control.
(ii) Based on the comprehensive voltage sensitivity model to determine the photovoltaic control area, the DDTW-FCM cluster partition algorithm is used to divide the power quality area and its dominant node, and four comprehensive power quality control areas of the AC and DC distribution network are determined.
(iii) Considering the power quality management function of PVMFGCI, the optimal compensation coefficients of power quality indicators in the four regions are solved based on its remaining capacity, which effectively improves the harmonics, reactive power, and imbalance of the AC side, and reduces the ripple of the DC bus wave, to achieve a more reasonable and economical AC and DC distribution network power quality collaborative governance.

## Figures and Tables

**Table 1:** Nodal Harmonic Pollution Data.

Node	5th Harmonic Current/A	7th Harmonic Current/A	11th Harmonic Current/A	13th Harmonic Current/A
2	12.3	8.1	5.7	3.3
4	13.7	6.6	4.2	2.5
7	16.7	9.3	6.5	4.1
8	8.4	5.1	3.4	1.1
11	9.8	4.7	3.5	0.4
13	15.6	9.1	5.8	1.3
15	16.9	7.6	3.1	2.6

**Table 2:** Photovoltaic Control Areas and Their Corresponding Nodes.

Area	Nodes with Photovoltaic Control Capability	Managed Nodes
R1	3	1, 2, 3
R2	7	6, 7, 8
R2	13	11, 12, 13, 14
R4	15	15, 16

**Table 3:** Results of Power Quality Area Division.

Area	Node for Regional Division	Dominant Node
I	1, 2, 3, 4, 5	3
II	6, 7, 8	7
III	9, 15, 16	15
IV	10, 11, 12, 13, 14	11

**Table 4:** Governance Optimization Results and Compensation Coefficients.

Area	Nodes with Photovoltaic Control Capability	Active Grid Connection/kW	Compensation Coefficient
I	3	15	(0.284, 0.170, 0.152)
II	7	15	(0.165, 0.201, 0.138)
III	13	15	(0.328, 0.272, 0.146)
IV	15	15	(0.406, 0.309, 0.167)

## Data Availability

Not applicable.

## Nomenclature

**Abbreviations**	
AC/DC	alternating current/direct current
PVMFGCI	photovoltaic multifunctional grid-connected inverter
DG	distributed generator
APF	active power filter
SVG	static var generator
UPQC	unified power quality conditioner
VSC	voltage source converter
DTW	dynamic time warping
ED	edit distance
DDTW	dynamic derivative time warping
**Variables**	
*U*_APF.h_, *I*_APF.h_	h-order harmonic compensation voltage/current vector produced by the photovoltaic grid-connected inverter
*m*_ij_	the harmonic control sensitivity of the photovoltaic grid-connected inverter from node *j* to node *i*
$\alpha_{hi}$	weight associated with the h-order harmonic voltage in node *i*
$E_{n_{h},ij}$, *S*_ij_, *B*_ij_	harmonic/reactive power/three-phase imbalance sensitivity
*W*_ij_	comprehensive voltage sensitivity
*VUF*_i_, $VUF_{i}^{\prime }$	the voltage imbalance degree of node *i* before/after compensation
*P*	the optimal number of clusters
*S*_h_, *S*_q_, *S*_2_	capacities of the DG grid-connected inverter for harmonic control, reactive power compensation, and three-phase imbalance control
$\varepsilon_{h}$ $,\;\varepsilon_{q}$ $,\;\varepsilon_{2}$	coefficients for harmonic control, reactive power compensation, and three-phase imbalance control of the remaining capacity of PVMFGCI
*I*_p1_, *I*_ph_, *I*_pq_, *I*_p2_	PVMFGCI output active current, harmonic wave control, reactive power compensation, and three-phase imbalance control current
$l_{h0}$ $,\;l_{q0}$ $,\;l_{20}$ $l_{h}$ $,\;l_{q}$ $,\;l_{2}$	harmonics, reactive power, and three-phase imbalance coefficients before compensation and after compensation
